# Human Stem Cell and Organoid Models to Advance Acute Kidney Injury Diagnostics and Therapeutics

**DOI:** 10.3390/ijms23137211

**Published:** 2022-06-29

**Authors:** Naomi Pode-Shakked, Prasad Devarajan

**Affiliations:** 1 Sackler Faculty of Medicine, Tel-Aviv University, Tel-Aviv 69978, Israel; naomi.podeshakked@cchmc.org; 2 Division of Nephrology and Hypertension, Cincinnati Children’s Hospital Medical Center, Department of Pediatrics, University of Cincinnati College of Medicine, Cincinnati, OH 45267, USA

**Keywords:** acute kidney injury, kidney organoids, kidney development, tubuloids

## Abstract

Acute kidney injury (AKI) is an increasingly common problem afflicting all ages, occurring in over 20% of non-critically ill hospitalized patients and >30% of children and >50% of adults in critical care units. AKI is associated with serious short-term and long-term consequences, and current therapeutic options are unsatisfactory. Large gaps remain in our understanding of human AKI pathobiology, which have hindered the discovery of novel diagnostics and therapeutics. Although animal models of AKI have been extensively studied, these differ significantly from human AKI in terms of molecular and cellular responses. In addition, animal models suffer from interspecies differences, high costs and ethical considerations. Static two-dimensional cell culture models of AKI also have limited utility since they have focused almost exclusively on hypoxic or cytotoxic injury to proximal tubules alone. An optimal AKI model would encompass several of the diverse specific cell types in the kidney that could be targets of injury. Second, it would resemble the human physiological milieu as closely as possible. Third, it would yield sensitive and measurable readouts that are directly applicable to the human condition. In this regard, the past two decades have seen a dramatic shift towards newer personalized human-based models to study human AKI. In this review, we provide recent developments using human stem cells, organoids, and in silico approaches to advance personalized AKI diagnostics and therapeutics.

## 1. The Unmet Need for More Personalized Human AKI Models

Acute kidney injury (AKI) is an increasingly common problem afflicting all ages, occurring in over 1 in 5 of non-critically ill hospitalized patients and in >30% of children and >50% of adults in critical care units. AKI is associated with serious short-term and long-term consequences [1,2].

AKI, defined as acute deterioration in Glomerular Filtration Rate (GFR), can be classified according to the nature of insult to the kidney, as: functional (previously designated as “pre-renal”), intrinsic (resulting from a multitude of injurious factors to kidney cells, including infections, medications, toxins, and prolonged ischemia) or obstructive (resulting from structural obstruction of urine flow) [3,4,5,6]. Of these, ‘intrinsic’ AKI represents direct kidney cell damage, while functional and obstructive AKI result from extra-renal causes with subsequent reduction of GFR. Persistence of either functional or obstructive AKI can result in kidney cell damage and progression to intrinsic AKI.

The most updated definition and staging of AKI are based on the Kidney Disease: Improving Global Outcomes (KDIGO) criteria and determined according to the degree of increase in serum creatinine from baseline or decrease in urine output [7]. AKI is an independent risk factor for mortality in critically ill patients and has been linked to subsequent progression to chronic kidney disease (CKD) [8,9]. Many efforts are directed toward improving prediction and prevention of AKI (e.g., identification of early biomarkers for AKI, and avoidance or reduced use of nephrotoxic medications), especially since treatments of AKI are currently limited to supportive care and kidney replacement therapy (KRT). At present, there are no targeted therapies for AKI, partly due to a lack of clinically relevant models to better understand the underlying pathophysiological mechanisms. Improved models could enable the development of targeted therapeutic strategies to prevent and treat AKI, attenuate its deterioration, and prevent progression to CKD.

Until recently, modeling of AKI for the discovery of novel diagnostics and therapeutics was primarily based on animal and cell culture models. Numerous animal models of AKI have been established, each with translational strengths, advantages, and challenges. However, murine models differ significantly from human AKI in terms of molecular and cellular responses, biomarkers, and clinical manifestations [10,11,12,13]. In addition, interspecies differences, high costs and ethical considerations related to animal models warrant a search for valid alternatives. Static two-dimensional cell culture models of AKI also have limited utility, since they have focused largely on hypoxic or cytotoxic injury to proximal tubules, and not on the several other nephron segments, vasculature, and interstitial cells that are also susceptible to AKI.

An optimal model of human AKI would harbor several key prerequisites. First, it would encompass the diverse specific cell types that could be targets of injury, based on their unique characteristics and function (each nephron segment expresses unique transporters and receptors that dictate its predisposition to injury and damage). Second, it would resemble the human physiological milieu as closely as possible, phenocopying the intricate cellular microenvironment, and cell-to-cell interactions. Third, it would yield sensitive and measurable readouts that are directly applicable to the human condition. In this regard, the past two decades have seen a dramatic paradigm shift, moving towards newer human-based models to study human disease. In this review, we provide recent developments using human stem cells, organoids, and in silico approaches to advance AKI diagnostics and therapeutics, complementing animal-based and cell culture models, and circumventing discrepancies stemming from inter-species differences.

## 2. Human Induced Pluripotent Stem Cell-Derived Kidney Organoids

Induced pluripotent stem cells (iPSCs) can be derived from any nucleated cell, hold infinite self-renewal potential, and can be differentiated into cell types of all germinal layers (i.e., endoderm, mesoderm and ectoderm). Accumulating knowledge regarding the complex process of nephrogenesis, including better understating of the reciprocal interaction between the ureteric bud and metanephric mesenchyme, has allowed recapitulation of these crucial developmental cues, in vitro. Consequently, iPSCs can be efficiently differentiated into human kidney organoids. These three-dimensional (3-D) multicellular structures encompass the diverse segments of the developing nephron and contain podocytes, proximal tubule, loop of Henle, and distal tubule cell types, as well as surrounding interstitial cells and even some vascular elements, thereby closely recapitulating normal kidney development [14,15,16,17,18,19,20,21]. PSC-derived kidney organoids mimic the intricate architecture of the developing kidney as well as some pivotal components of its physiology. For instance, iPSC-derived kidney organoids have been shown to exhibit albumin and dextran endocytosis by the proximal tubules [20].

Thus, kidney organoids represent a model system for assessing patient-specific kidney cell susceptibility to diverse AKI culprits (such as hypoxia, drugs, toxins, infections, and inflammatory cytokines) as well as evaluation of their potential regenerative capacity. Moreover, analysis of cell-specific responses to injury at a genomic, transcriptomic, epigenetic and proteomic levels throughout the continuum of kidney cell damage, offers insights for the identification of novel mechanisms and biomarkers highly sensitive for all phases of kidney cell injury and repair. Reproducible protocols have now been optimized, enabling the directed differentiation of iPSCs into kidney organoids with progressively improved efficiency, maturation, diverse nephron segment representation, and even vascularization [22,23,24,25,26,27,28,29].

**Figure 1:**
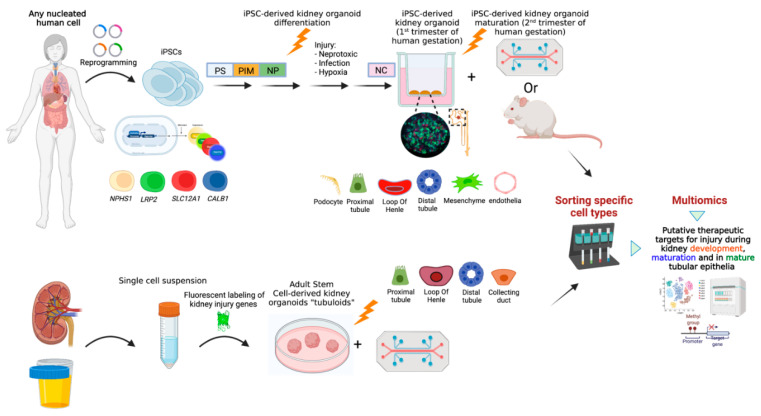
Kidney organoids as a useful tool for uncovering pathomechanisms underlying different types of Acute Kidney Injury and unveiling putative personalized therapeutic targets. Induced pluripotent stem cells (iPSCs) can be reprogrammed from any somatic nucleated cell. These iPSCs can undergo sequential differentiation toward kidney organoids. The use of CRISPR/Cas9 gene-edited reporter iPSC lines in which a fluorescent marker is inserted under the promotor of a specific kidney-cell lineage gene (i.e., *NPHS1* for podocytes, *LRP2* for PT, *SLC12A1* for LOH, *CALB1* for DT, etc.), enables a lineage tracing system during kidney organoid differentiation, maturation and following injury. Maturation of kidney organoids equivalent to the second trimester of human gestation can be achieved via transplantation of kidney organoids under the renal capsule of immunodeficient mice or via growing them on a chip under continuous flow (upper panel); Organoids composed of the different epithelial cells that comprise the kidney tubules (also known as ‘tubuloids’) can be generated either from mature kidney tissue or from urine (lower panel). Following a processing procedure, a single cell suspension is achieved. Cells are subsequently aggregated and cultured in specific conditions. Tubuloids can grow on scaffolds (‘chip’) that enable delivery of nephrotoxins. Epithelial tubuloids are amenable to genetic editing. Fluorescent labeling of kidney injury genes enables analysis of response to injury at a cell-specific manner. Fluorescent based cell sorting of post-injury kidney organoids and tubuloids followed by genomic, transcriptomic, proteomic, metabolomic and epigenomic analyses may unveil unique effects of different types of injuries on specific kidney cell types, signaling pathways and molecular components and bring forward putative AKI-pathomechanism-driven therapeutic targets. PT, Proximal Tubules; LOH, Loop of Henle; DT, Distal Tubules; PS, Primitive Streak; PIM, Posterior Intermediate Mesoderm; NP, Nephron Progenitors; NC, Nephron cells.

Kidney organoids have been successfully used for modelling of AKI induced by several nephrotoxic drugs as well as infectious pathogens with known renal cell-specific effects and have yielded unexpected findings (Figure 1). For example, a recent study demonstrated gentamycin induced cell specific injury in iPSC-derived kidney organoids affecting both proximal and distal tubule cells 18]. This was demonstrated via co-staining of the Kidney Injury Molecule-1 (KIM-1, an established marker of proximal tubule damage following AKI), along with the DNA damage marker γH2AX with either the proximal tubule marker LTL, the distal tubule marker CDH1, or the podocyte marker PODXL. This pioneering study was the first to establish the use of human kidney organoids for the study of nephrotoxin-induced AKI. Another study in human iPSC-derived kidney organoids demonstrated the novel finding that cisplatin induces not only proximal tubule injury but also interstitial cell death, via colocalization of the DNA damage marker γH2AX with an interstitial cell marker MEIS1 [21]. Furthermore, the authors found that cisplatin administration in low repeated doses resulted in cumulative damage along with predominant activation of the TGF beta pathway, suggestive of mimicking the transition from AKI to CKD [21]. Several groups have now developed protocols for massive, reproducible production of iPSC-derived kidney organoids via automated systems combined with bioprinting, enabling high-throughput screening for nephrotoxicity and prediction of safety and efficacy.

Kidney organoids have recently been utilized to study infectivity of the SARS-CoV-2 virus and its neutralization by soluble Angiotensin Converting Enzyme 2 (ACE2) protein [30,31]. The investigators analyzed the capacity of a newly bioengineered soluble ACE2 (sACE2) compound to inhibit infection of human embryonal stem cell (hESC)-derived kidney organoids by the SARS-CoV-2 virus. They concluded that hESC-derived kidney organoids can be infected by SARS-CoV-2 via the known route of infection and that the new sACE2 effectively inhibits their infection.

Until recently, lack of patent vascular system and cellular immaturity in 3-D kidney organoids restricted their application mainly to the study of AKI as a result of drug-induced nephrotoxicity or for modeling ischemic kidney injury as well as insult to mature kidney cells. Recent efforts at enhanced maturity and vascularization of PSC-derived kidney organoids have increased their value as in vitro models for AKI. Implantation of kidney organoids under the kidney capsule of immunodeficient mice results in formation of a vascular network, perfusion of glomeruli, and enhanced maturation and polarization of nephron structures [32]. This was recapitulated in vitro via culturing of kidney organoids on millifluidic chips, suggesting a crucial role for fluidic shear stress on both vascularization and nephron maturation [25]. These studies have paved the path for in vitro models of AKI induced by circulatory failure. Improved maturation of these kidney organoids including vascularization further enhances their clinical relevance. Several groups have now established methods for generation of iPSC-derived organoids specifically of the developing and mature collecting system [26,33]. Uchimura et al. developed a protocol that improved maturation of the PT cells as well as induction of intercalated and principal cells of the CD via aldosterone and/or vasopressin [34]. Utilizing this protocol, they were able to show that both Neutrophil gelatinase-associated lipocalin (NGAL) which is an early biomarker for ischemic, septic, or nephrotoxic kidney injury, expressed in both thick ascending limb and collecting duct and HAVCR1, a biomarker for PT injury, were upregulated following treatment with cisplatin in a cell specific manner.

From a kidney regeneration standpoint, subcapsular administration of iPSC-derived kidney organoids to mice with AKI induced by ischemia/reperfusion resulted in significant reduction in BUN and creatinine and attenuated adverse histopathological changes [35]. The investigators generated an OSR1-GFP/SIX2-tdTomato double knock-in reporter hiPSC line which enabled sorting of nephron progenitor cells (NPS; i.e., OSR1+SIX2+) from hiPSC following several stages of differentiation. These (along with their negative counterparts: OSR1+SIX2−, OSR1-SIX2+ and OSR1-SIX2−) were transplanted under the kidney capsule of immunodeficient mice following induction of ischemia/reperfusion (I/R) resulting in AKI. Following I/R and transplantation, serum creatinine and BUN were measured at several time points demonstrating an initial decrease (at the 2-h point) in the NPC compared to iPSC or saline transplantation followed by gradual equalization of levels after 12 h. Moreover, histopathological scores for tubular necrosis, tubular dilatation, loss of tubular borders and interstitial fibrosis were significantly improved on day 12 after I/R and transplantation of SIX2+OSR1+ NPCs compared to iPSC or saline. 

## 3. Human Adult Stem Cell-Derived Kidney Tubuloids

Kidney tubular epithelial organoids, termed “tubuloids”, are complex multicellular structures that mimic the architecture and functionality of the different epithelial subpopulations comprising the mature nephron [36]. They express characteristic markers of all specific tubular segments and display specific tubule segment functions. Tubuloids have been successfully generated from adult human kidney tissue (i.e., renal biopsies, nephrectomies) [37] and even from urine [36,38]. The ability to use urine, a non-invasive, easily accessible and unlimited source, as an alternative to kidney tissue for reliable tubuloid generation, holds promise for research and clinical applications. Kidney tubuloids have been used to study normal kidney physiology, nephrotoxicity, drug testing, infections, and to model kidney diseases [39,40,41] (Figure 1). While tubuloids do not entirely recapitulate the complexity of their tissue of origin to the same extent as iPSC-derived kidney organoids, they possess several advantages. They can be generated without need for genetic modification, are less labor-intensive, maintain genomic stability, represent an in vitro mature replica of human tubular nephron and are devoid of the ethical considerations that accompany PSCs.

Recently, mature kidney-derived 3-D organoids were generated from adult kidney tissue demonstrating expression of markers of glomerular podocytes (i.e., Podocin, Synaptopodin, and Nephrin) concomitant with markers of tubular epithelia aquaporin-1 (AQP1) and collecting duct (AQP-3). Functionally, these organoids produced EPO in response to induced hypoxia [37]. Moreover, treatment of these 3-D organoids with aspirin, penicillin G or cisplatin resulted in increased GGT activity and upregulation of KIM-1, suggestive of acute proximal tubule injury [37]. Tubule cell infection with BK nephropathy was recapitulated in kidney tubuloids infected by the virus as manifested by similar timing of histological and gene expression patterns to those seen in the clinical setting [28]. Adult kidney spheroids as well as primary human kidney monolayer cultures infected with SARS-CoV-2 virus display a specific trophism towards kidney cells and increased expression of cell damage markers in infected tubule cells [42]. 

## 4. Human Kidney Tumor-Derived Stem Cells

In vitro personalized models for human neoplasms provide insights into the unique cellular, molecular and functional complexity of tumors for development of targeted treatments. A reliable model should recreate the intricate interaction between the 3-D tumor niche and cancer cells to overcome the intrinsic limitations of previous 2-D cancer cell cultures. Tumor-derived organoids that mimic the cellular and genetic heterogeneity of their parental tumor of origin in vitro over time have been successfully established for numerous malignancies, demonstrating high predictive value for patient specific drug responses. In some instances, tumor cells naturally repeat the consecutive stages of embryonic development resulting in formation of 3-D spheroids/organoids that recapitulate the cellular complexity of their parental tumor. This is particularly relevant to a subgroup of pediatric solid tumors which are thought to be embryonal in origin, displaying morphological and molecular characteristics of a developmental process gone awry.

The paradigm for such tumors is Wilms’ tumor (WT), the most common pediatric renal malignancy, composed of undifferentiated blastema and more differentiated epithelia and stroma equivalent to the metanephric mesenchyme, tubular epithelia and interstitium found in the developing kidney. WT patient-specific organoids contain the same cellular elements found in their parental tumor, recapturing its heterogeneity in vitro [43]. Three-dimensional spheroid formation has been successfully demonstrated from high risk blastemal-predominant WT as well as epithelial and stromal WT [44]. The organoids corresponded to the primary tumor of origin in both histology and gene expression, were susceptible to genetic modifications and were able to generate WT patient-derived xenografts when injected to immunodeficient mice. Pediatric kidney tumor organoid biobanks have now been established, consisting of matched tumor organoids and normal tubuloids from 50 individual patients with four different kidney neoplasms (i.e., WT, rhabdoid tumor, renal cell carcinoma, and congenital mesoblastic nephroma). Using high resolution 3-D imaging, whole-genome DNA sequencing, single cell RNA-sequencing and DNA methylation analyses, WT patient-specific organoids were shown to be composed of the same cellular elements found in their parental tumor, recapturing its heterogeneity in vitro. Combined with their matching normal kidney tubuloids, this unique biobank paves the path for identification of personalized targeted therapies against these tumors that are less nephrotoxic as their effects can be concomitantly evaluated on both patient-derived malignant and normal organoids, thus limiting chemotherapy induced AKI.

## 5. Potential Role of Gene Editing in Human Kidney Organoids and Tubuloids

The past decade has introduced the CRISPR (Clustered Regularly Interspaced Short Palindromic Repeats)/Cas (CRISPR-associated protein) gene editing system, which has provided a major leap toward our understanding and control of the human genome. Of the multitude of genome editing approaches, the CRISPR/Cas is probably the most rapid, accurate and efficient. The most prevalent CRISPR/Cas system used for gene editing of human cells is the one utilizing the Cas9 endonuclease. The basics of this system have been recently reviewed [45]. Briefly, adapted from a genome editing system in bacteria serving as an immune system against infecting viruses, the CRISPR-Cas9 complex is utilized to edit the human genome via generation of small RNA fragments (guide RNA) that contain a short sequence that recognizes and binds to a specific target sequence of DNA as well as to the Cas9 enzyme. Consequently, the guide RNA recognizes the specific DNA sequence, and the Cas9 endonuclease performs a double strand break at the targeted location. Following this double strand break, the cell native DNA repair machinery is utilized to make changes to the distinct DNA sequence (gene knock-in, gene knock-out, replace the existing sequence with a customized DNA sequence or generate gene fusions).

The concomitant evolution of in vitro, human stem cell based, culturing methods that recapitulate many of the complex characteristics of the in vivo tissue/organ (i.e., organoids) offers a unique opportunity for combining these techniques. Indeed, CRISPR/Cas genomic editing has been successfully used to study gene function, human development and model human diseases in both iPSC-derived organoids (either 2-D or 3-D) and in tissue-derived somatic stem cells 3-D organoids [45].

CRISPR/Cas mediated genomic editing has been applied for modeling genetic kidney diseases in patient-derived iPSC derived kidney organoids. These kidney organoids recapitulate the disease phenotype in the organoids (i.e., cyst generation in AD/AR polycystic kidney diseases—mutations in PKD1/2 or PKHD1, respectively; foot processes effacement in congenital nephrotic syndrome due to mutation in the nephrin gene, among others) and following CRISPR/Cas mediated correction of the patient mutation in the iPSCs, the phenotype is reverted [17,24].

In the context of AKI, CRISPR technology has recently been used to generate kidney lineage-specific fluorescent reporter iPSC lines that enable zooming in and sorting out specific kidney cell types following differentiation into kidney organoids [46,47,48]. These facilitate tracing of different mechanisms of injury to specific cell types and allow sorting out distinct cell populations for high resolution genomic, proteomic, epigenomic and metabolomic characterization at the single cell level (Figure 1). 

One attractive future direction on which this technique may shed light, is that of neonatal and specifically preterm AKI. AKI prevalence in the neonatal intensive care unit (NICU) is extremely high and is associated with high mortality rates [49,50,51]. Advancements in modern medicine have resulted in increased survival of extreme preterm infants (<28 weeks of gestation) who are born with lower nephron endowment and consequently lower kidney reserve. These baseline characteristics combined with common cardiovascular abnormalities (e.g., Patent Ductus Arteriosus), high exposure to nephrotoxic medications, and poor nutrition, greatly increase their renal vulnerability and consequently their risk for development of AKI, estimated to be as high as 25–48% in recent cohorts [49,50,51,52,53,54]. iPSC-derived kidney organoids, which can reach a degree of maturation equivalent to kidneys at the second trimester of human gestation, in congruence with cell specific CRISPR/Cas induced reporters, offer a unique window into the specific effects of insults to the developing human kidney and may reveal potential personalized targets for therapeutic intervention and kidney regeneration in this highly vulnerable patient population. For example, HMOX1 is a known marker of oxidative stress. Recently, investigators generated an iPSC HMOX1 reporter line, differentiated it into kidney organoids, and used this line for screening of a panel of blind-coded compounds for putative nephrotoxicants [55]. 

## 6. Kidney on a Chip

The advent of microfluidic organ-on-a-chip technologies has dramatically re-envisioned in vitro modeling of kidney injury [56,57]. Kidney-on-a-chip models are perfusable microfluidic devices on which renal cells are seeded on bio-printed 3-D chips, allowing media to flow across the cell surface [58,59]. The addition of a vascular component, which can be perfused, more accurately simulates physiological conditions of the kidney 3-D microenvironment [60]. While primary proximal tubule cells have most commonly been used in microfluidic model systems, there have also been successful endeavors seeding other types of renal cells, such as iPSC-derived podocytes co-cultured with human glomerular endothelial cells to create glomerulus-on-a-chip systems [61,62]. Most recently, kidney-on-a-chip models have been established using multiple cell populations from single donors [63], demonstrating their potential use in personalized medicine, as well as coupled with other organs-on-a-chip, as part of multi-organ models [64]. While the main focus of most studies utilizing kidney-on-a-chip technology thus far has been nephrotoxicity from drugs such as cisplatin, tenofovir, tobramycin and cyclosporin A [55,56,57], they also provide a unique opportunity to model AKI in a wide variety of additional etiologies (e.g., ischemia, hypoxia).

## 7. Possible Application of Kidney Organoid Models for Prevention of AKI-CKD Progression

The ability to model all human kidney cells in vitro not only allows zooming in on the molecular pathways involved in response to injury, but also opens a window for identification of treatments that can reverse this response. 

Recent studies in mice models of AKI utilized single cell RNA sequencing to unveil unique molecular pathways involved in response to acute kidney injury [65,66]. For example, Kirita et al. identified novel proximal tubule cell subpopulations in response to acute kidney injury which informed on deterioration toward chronic kidney damage versus complete recovery [65]. 

Translating these findings into a human organoid model of kidney injury, Gupta el al. utilized iPSC-derived kidney organoids to focus on the pathomechanism and characteristics of transition from intrinsic to incomplete cellular repair in response to kidney injury induced by cisplatin [29]. Using the kidney organoid system, they demonstrated that a single exposure to cisplatin results in complete repair (designated by the authors as “intrinsic repair”) which is consistent with in vivo models and involves proximal tubule dedifferentiation and entry into cell cycle. Additionally, injured proximal tubule cells (LTL+) demonstrated upregulation in DNA damage marker γH2AX and the Homology-Directed Repair (HDR) protein, Fanconi anemia complementation group D2 (FANCD2) compared to untreated controls. However, 7 days after cisplatin exposure, LTL+ cells showed reduction in γH2AX and reduction of cell cycle activity with concomitant preservation of normal tubular cell morphology. In contrast, recurrent exposure to low doses of cisplatin leads to an incomplete repair manifesting histologically with increased tubular atrophy, reduction of tubule number and increased collagen deposition in the kidney organoids. At the molecular level, transition from intrinsic to incomplete repair was accompanied by a significant downregulation of HDR gene expression including FANCD2 and RAD51 recombinase (RAD51). Moreover, FANCD2 expression was inversely correlated with degree of kidney fibrosis, not only in the kidney organoids but also in kidney biopsies from diabetic kidney disease patients. Finally, rescue of FANCD2 via treatment of kidney organoids exposed to repeated cisplatin doses with the DNA ligase IV inhibitor, SCR7 resulted in limited transition from intrinsic to incomplete repair, suggesting targeting of FANCD2/RAD51 pathway for halting disease progression in the AKI-CKD transition [29].

## 8. Conclusions

Current treatments for AKI include medical supportive care or kidney replacement therapy (KRT) and are not directed at the pathomechanisms underlying individual kidney cell response to injury. Utilizing currently available human-based kidney organoid models for multiomics analysis of specific kidney cell response to injury, holds tremendous potential for unveiling novel therapeutic targets for AKI. The next few years will witness a leap toward personalized, pathomechanism-guided approaches for diagnosis and treatment of AKI, as well as for prevention of AKI-to-CKD progression. As the major drawback of in vitro organoid-based models of AKI is the lack of a systemic context, limiting the types of kidney injury which can be recapitulated, combining kidney organoids with micro-fluidic chips or implantation into murine models will be a promising next step for leveraging the accuracy of these models to mimic human kidney physiology and pathology [67,68,69].

## Data Availability

Not applicable.
